# Thyroid Steal Syndrome Secondary to Active Hyperthyroid State

**DOI:** 10.7759/cureus.22529

**Published:** 2022-02-23

**Authors:** Harrison Humphries, Johnathon Chung, Reza Pirzadeh, Wesley Jones, Mohamad Ezzeldin

**Affiliations:** 1 Internal Medicine, HCA Houston Healthcare Kingwood/University of Houston College of Medicine, Kingwood, USA; 2 Neurological Surgery, HCA Houston Healthcare Kingwood/University of Houston College of Medicine, Kingwood, USA; 3 Interventional Neuroradiology, HCA Houston Healthcare Kingwood/University of Houston College of Medicine, Kingwood, USA

**Keywords:** ct head angiogram, digital subtraction angiography(dsa), ct angiogram, thyroid steal syndrome, conventional cerebral angiogram, tia, hyperthyroidism, thyroid steal

## Abstract

Thyroid steal syndrome (TSS) is a rare condition characterized by recurrent transient ischemic attack (TIA) that is found to be due to a large thyroid goiter or thyroid hormone derangement causing a diversion of blood flow from the cerebral circulation. Here we report a patient with a history of multiple TIAs thought initially to be due to intracranial arterial stenosis based on CT angiography (CTA) findings, but later found to be secondary to hyperthyroid state causing TSS. To our knowledge, this is the first-ever reported case of TSS secondary to hyperthyroidism and only the second case of TSS secondary to any thyroid hormone derangement.

## Introduction

Most transient ischemic attacks (TIAs) are the result of blockage or narrowing within the intracranial blood vessels. However, deviation of blood from the cerebral circulation due to pathology involving extracranial blood vessels can also result in TIA. This is the case for the rare condition known as thyroid steal syndrome (TSS). TSS is a condition characterized by episodes of transient cerebral ischemia that occur secondary to the “steal,” or diversion, of blood from the brain to the thyroid due to thyroid goiter and, less commonly, thyroid hormone derangements [1]. Patients with this condition commonly present with a history of multiple TIAs and are found on angiography to have enlargement of the thyroid arteries in the setting of a voluminous goiter or thyroid hormone derangement [1-3]. Only four cases of TSS have been clearly documented in the literature, three of which were secondary to thyroid goiter [1-3]. In this case report, we present a rare case of TSS secondary to an active hyperthyroid state, the likes of which have never previously been documented in the literature.

## Case presentation

**Figure 1:**
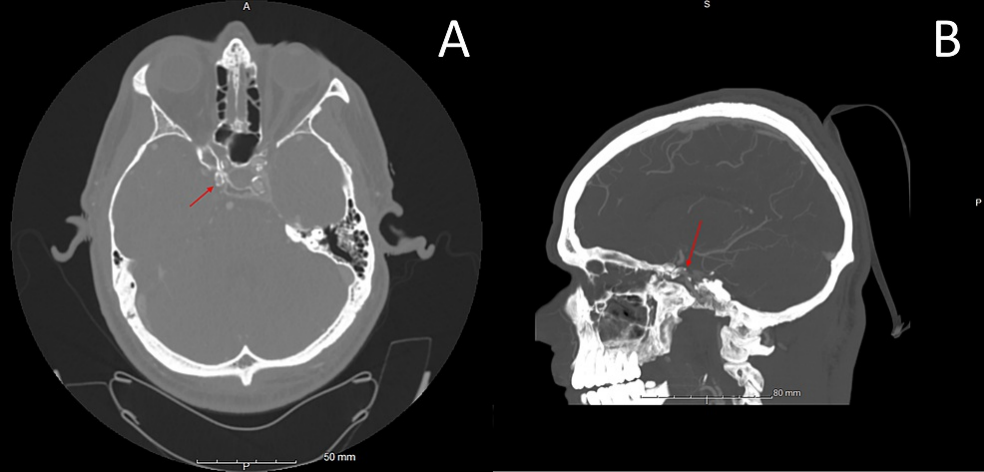
CT angiogram of the head, axial cut (A) and sagittal cut (B) demonstrating atherosclerotic changes in the right clinoid ICA (red arrow) resulting in approximately 70% stenosis per WASID criteria. CT: computed tomography; ICA: internal carotid artery; WASID: Warfarin-Aspirin Symptomatic Intracranial Disease

A 45-year-old male with a past medical history of insulin-dependent diabetes mellitus and peripheral artery disease presented to the emergency department with complaints of two episodes of left hemiparesthesia and hemiplegia in the last three months. The patient reported that the first episode lasted 30-40 minutes and the second lasted two hours. On arrival at the emergency department, the patient was found to be tachycardic with a heart rate of 129 and hypertensive with a blood pressure of 139/77. There were no focal neurological deficits on the initial physical exam. Initial laboratory examination revealed a blood glucose of 505, thyroid-stimulating hormone (TSH) of <0.015 mIU/L (normal range: 0.465-4.68 mIU/L), free T4 of 4.52 ng/dL (normal range: 0.78-2.19 ng/dL), thyrotropin receptor antibody of 4.64 IU/L (normal range: 0.00-1.75 IU/L), and thyroid microsomal antibody of 299 IU/mL (normal range: 0-34 IU/mL). Initial computed tomography (CT) scan of the head without contrast showed no acute intracranial abnormality. CT angiogram (CTA) of the head reported no intracranial large vessel occlusion, but CTA of the neck reported greater than 70% stenosis of the right clinoid internal carotid artery (ICA), up to 50% stenosis of the left petrous, cavernous, and clinoid ICA, and greater than 50% stenosis of the intradural right vertebral artery (Figure 1).

**Figure 2:**
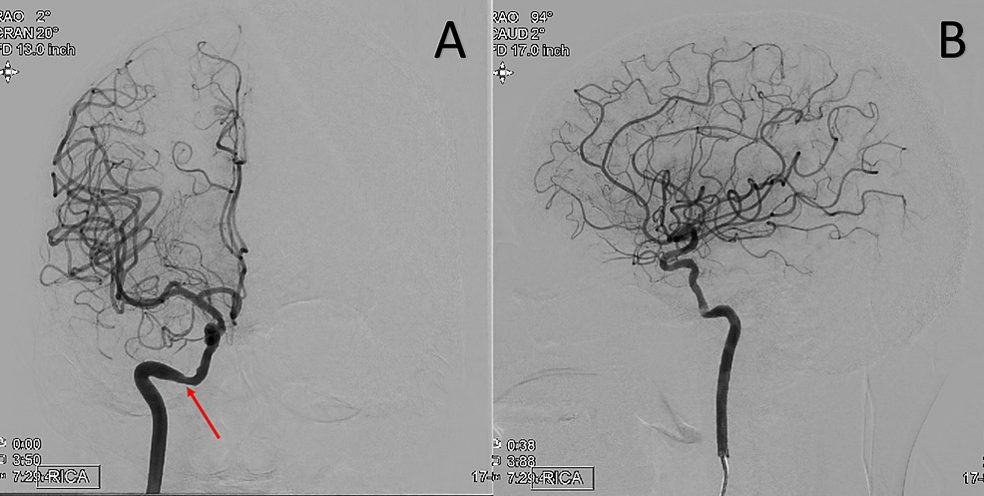
Right internal carotid artery angiogram, AP view (A) and lateral view (B) demonstrating atherosclerotic changes at the right lacerum ICA (red arrow) with no hemodynamically significant flow limiting stenosis in the clinoid segment. AP: anterior-posterior; ICA: internal carotid artery

Follow-up MRI of the brain revealed no acute intracranial abnormality. An echocardiogram with bubble study was performed and revealed an ejection fraction of 60%-64% with no evidence of patent foramen ovale. The patient's TIAs were initially attributed to symptomatic right intracranial ICA stenosis. Cerebral digital subtraction angiography (DSA) demonstrated mild atherosclerotic changes but ruled out significant flow limiting extra or intracranial stenosis (Figure 2).

**Figure 3:**
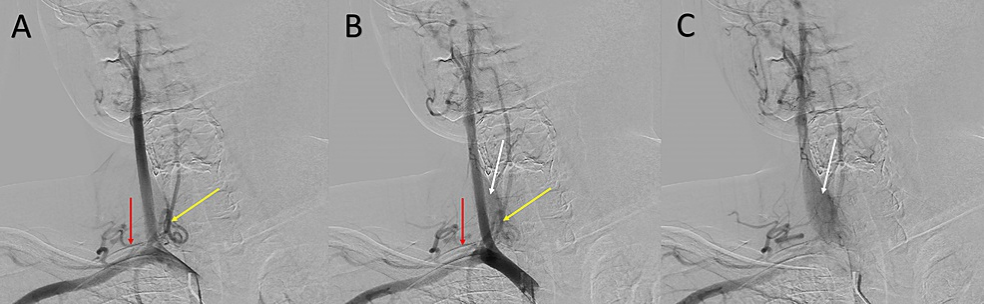
Brachiocephalic artery angiogram progressing from A to C, AP view, demonstrating prominent right thyrocervical trunk (red arrow), right inferior thyroid artery (yellow arrow) and thyroid blush (white arrow). AP: anterior-posterior

**Figure 4:**
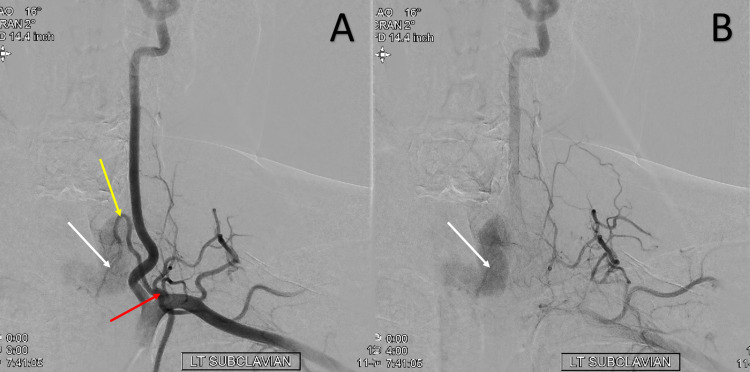
Left subclavian artery angiogram progressing from A to B, AP view, demonstrating significantly enlarged left thyrocervical trunk (red arrow), left inferior thyroid artery (yellow arrow) and thyroid blush (white arrow). AP: anterior-posterior

However, the study revealed prominent bilateral thyrocervical trunks with a prominent vascular blush of the thyroid gland (Figures 3-4). These findings paired with laboratory findings indicating hyperthyroidism led to the consideration of the diagnosis of TSS. The patient was initiated on methimazole and propranolol for treatment of hyperthyroidism and was discharged with a plan to follow up with endocrinology.

## Discussion

TSS was first described in 1982 in a patient who presented with TIA after treatment of hyperthyroidism rendered the patient hypothyroid. The patient was found to have significant dilation of the arteries supplying the thyroid on cerebral angiogram. After cessation of antithyroid medications, the patient was rendered euthyroid and the symptoms of TIA ceased [1]. In this case, it was theorized that the increased thyroid blood flow was stimulated by the high levels of TSH (>100 mIU/ml) [1,4]. The other three documented cases of TSS were all in patients who presented with recurrent TIAs and were found to have a voluminous thyroid goiter [2,3]. In all three of these cases, the arteries supplying the thyroid were found to be enlarged by cerebral angiogram and surgical removal of the thyroid resulted in long-term prevention of symptom recurrence [2,3]. In these cases, it was hypothesized that the development of a voluminous goiter resulted in the increased dilation of thyroid arteries and blood flow to the goiter which resulted in compromised carotid artery flow [2,3].

To our knowledge, we report the first case of TSS that is the result of an active hyperthyroid state, and only the second case of TSS that is the result of any thyroid hormone derangement rather than thyroid goiter. Contrary to prior reported cases of TSS, in our case, the increased thyroid blood flow and vascularity that resulted in the steal phenomenon did not occur due to a high TSH level or a thyroid goiter, as our patient had neither. The patient’s high levels of thyrotropin receptor antibody likely explain the increased thyroid vascularity and blood flow, as these antibodies are able to mimic the effect of TSH [5].

Another component that distinguishes this case from prior TSS cases is the utilization of CTA head and neck prior to cerebral DSA. Prior reported cases of TSS presented for initial evaluation from 1977 to 1986 [1-3]. As the first CTA was not performed until 1992, CTA was not an option at the time [6]. In this case, CTA head and neck reported >70% right ICA atherosclerotic cavernous/supraclinoid segment stenosis which was not substantiated by follow-up cerebral DSA (Figures 1-2). While CTA is widely considered an accurate, less invasive tool in estimating the degree of stenosis in the carotid arteries, more recent studies comparing CTA and DSA have demonstrated carotid stenosis misestimation by CTA [7]. One such study demonstrated that out of 90 patients with significant carotid stenosis on CTA, only 70 patients were subsequently found to have significant carotid stenosis on DSA [7]. While there was strong agreement between CTA and DSA when CTA estimated carotid stenosis to be >90%, the agreement was much weaker when CTA estimated carotid stenosis to be 50%-70% [7]. 

TSS should be considered in the context of any patient presenting with transient neurological symptoms who are found to have a large thyroid goiter on exam or laboratory evidence of thyroid hormone derangement. It is important to consider TSS as the possible etiology in patients with recurrent TIAs even after CTA demonstration of carotid stenosis, as CTA can misestimate carotid stenosis especially when the reported stenosis is 50-70% [7]. Correctly making the diagnosis of TSS is crucial, as the condition is treated by addressing the underlying cause. This means surgical intervention in the case of a thyroid goiter and medical management in the case of thyroid derangements [1-3].

## Conclusions

Here we present the first reported case of TSS secondary to a hyperthyroid state and only the second case of TSS due to a thyroid hormone derangement. The diagnosis of TSS should be considered in all patients with recurrent TIA and concurrent goiter and/or thyroid hormone derangement. This case highlights the importance of being aware of this entity, as there is a potential for CTA findings to misguide the clinician towards other etiologies.
